# Two different strategies of *Diversispora spurca*-inoculated walnut seedlings to improve leaf P acquisition at low and moderate P levels

**DOI:** 10.3389/fpls.2023.1140467

**Published:** 2023-02-23

**Authors:** Ying-Ning Zou, Yong-Jie Xu, Rui-Cheng Liu, Guang-Ming Huang, Kamil Kuča, Anoop Kumar Srivastava, Abeer Hashem, Elsayed Fathi Abd_Allah, Qiang-Sheng Wu

**Affiliations:** ^1^ Tibet Plateau Walnut Industry Research Institute, College of Horticulture and Gardening, Yangtze University, Jingzhou, Hubei, China; ^2^ Hubei Academy of Foresty, Wuhan, China; ^3^ Department of Chemistry, Faculty of Science, University of Hradec Kralove, Hradec Kralove, Czechia; ^4^ ICAR-Central Citrus Research Institute, Nagpur, Maharashtra, India; ^5^ Botany and Microbiology Department, College of Science, King Saud University, Riyadh, Saudi Arabia; ^6^ Plant Production Department, College of Food and Agricultural Sciences, King Saud University, Riyadh, Saudi Arabia

**Keywords:** mycorrhiza, P deficit, phosphate transporter, purple acid phosphatase, walnut

## Abstract

Walnut (*Juglans regia*) is an important nut tree species in the world, whereas walnut trees often face inadequate phosphorus (P) levels of soil, negatively limiting its growth and yield. Arbuscular mycorrhizal fungi (AMF) can colonize walnut roots, but whether and how AMF promotes walnut growth, physiological activities, and P acquisition is unclear. The present study aimed to evaluate the effects of *Diversispora spurca* on plant growth, chlorophyll component concentrations, leaf gas exchange, sugar and P concentrations, and expression of *purple acid phosphatase* (*PAP*) and *phosphate transporter* (*PT*) genes in leaves of *J*. *regia* var. Liaohe 1 seedling under moderate (100 μmol/L P) and low P (1 μmol/L P) levels conditions. Three months after inoculation, the root mycorrhizal colonization rate and soil hyphal length were 45.6−53.2% and 18.7−39.9 cm/g soil, respectively, and low P treatment significantly increased both root mycorrhizal colonization rate and soil hyphal length. Low P levels inhibited plant growth (height, stem diameter, and total biomass) and leaf gas exchange (photosynthetic rate, transpiration rate and stomatal conductance), while AMF colonization significantly increased these variables at moderate and low P levels. Low P treatment limited the level of chlorophyll *a*, but AMF colonization did not significantly affect the level of chlorophyll components, independent on soil P levels. AMF colonization also increased leaf glucose at appropriate P levels and leaf fructose at low P levels than non-AMF treatment. AMF colonization significantly increased leaf P concentration by 21.0−26.2% than non-AMF colonization at low and moderate P levels. Low P treatment reduced the expression of leaf *JrPAP10*, *JrPAP12*, and *JrPT3;2* in the inoculated plants, whereas AMF colonization up-regulated the expression of leaf *JrPAP10*, *JrPAP12*, and *JrPT3;2* at moderate P levels, although AMF did not significantly alter the expression of *JrPAPs* and *JrPTs* at low P levels. It is concluded that AMF improved plant growth, leaf gas exchange, and P acquisition of walnut seedlings at different P levels, where mycorrhizal promotion of P acquisition was dominated by direct mycorrhizal involvement in P uptake at low P levels, while up-regulation of host *PAPs* and *PTs* expressions at moderate P levels.

## Introduction

Phosphorus (P) is an important nutrient for plant growth and productivity. However, in the soil, P is immobilized in the form of aluminum/iron or calcium/magnesium phosphate (Pi), which prevents it from being acquired by plants. In addition, the utilization rate of Pi in agriculture is 15−20%, making crops often faced with P starvation (Malhotra et al., 2018). The use of a large amount of P fertilizers leads to the decline in soil physical and chemical properties, pollutes soil and aquatic environment, changes biodiversity, and triggering significant carbon emissions (Li et al., 2022). To deal with P starvation, plant roots absorb Pi directly from the soil by Pi transporters (PTs) or indirectly through their symbiotic arbuscular mycorrhizal fungi (AMF) (Wang et al., 2021). The inorganic Pi absorbed by the roots is loaded through the Casparian band in the xylem vessels and then transferred to the shoots *via* the protein, phophate 1 (Sandhu and Rouached, 2022).

Attempts have also been made to use some beneficial microorganisms to enhance plant growth under P starvation conditions, such as the AMF (Adeyemi et al., 2021). AMF is an ancient class of fungi that establish mycorrhizal symbiosis on about 80% of terrestrial plants, where AMF obtains their required sugars and fatty acids from the plant partners in exchange for the acquired P (Smith and Smith, 2011). In addition, mycorrhizal extraradical mycelium can extend beyond the Pi depletion zone in the rhizosphere to absorb Pi that cannot be obtained by plants, increase the area of root absorption area, release organic acids such as citrate and malate to dissolve organic Pi, and activate expressions of specific *PT* genes in both mycorrhizal roots and AMF to absorb and transfer Pi, thus enhancing plant P acquisition (Etesami et al., 2021). AMF also changes Pi absorption kinetic parameters and stimulates other microbial activities in the soil to jointly promote P absorption of host plants (Smith et al., 2015). In addition to *PTs*, *purple acid phosphatase* (*PAP*) genes are also involved in mycorrhizal enhancement of host P acquisition. In soybeans, overexpression of *GmPAP33* resulted in increased plant P concentrations after AMF inoculation and also participated in arbuscule degradation (Li et al., 2019).

Walnut (*Juglans regia* L.) is an important nut tree species in the world, cultivated in Asia, North America, Europe and South America, of which China is the world’s largest walnut producer, with 7.8 million hm^2^ of walnut planted area and a yield of 4.806 million t in 2020 (Ma and Ning, 2021). However, the soil fertility of walnut orchards in China is relatively low, especially the Olsen-P level, resulting in low fruit yield and oil content (Zhang, 2014), which seriously limits the high yield and quality of walnut. In addition, in the United States, many walnut trees in Lake County, California also suffer from P starvation, mainly in the volcanic soil (Serr, 1960), resulting in small and thin leaves, thin and few branches, and purplish red in the petiole and leaf backside in severe cases. Some AMF populations have been found to inhabit the rhizosphere of walnuts and confer many important physiological contributions to the host walnut (Ma et al., 2021). Mao et al. (2022) observed that the AMF colonization rate of walnut roots in Yunnan, China ranged from 75.67% to 84.37%, along with the genus *Acaulospora*, *Diversispora*, *Funneliformis*, *Glomus*, *Rhizophagus*, *Scuteiiospora*, *Sclerocystis*, and *Septoglomus* recorded in the rhizosphere. And, further inoculation revealed that these native AMF species significantly increased leaf and root P content (Mao et al., 2022). In walnut seedlings inoculated with five AMF species, Huang et al. (2020) observed improved plant growth, coupled with the best effect on *D*. *spurca*. Mycorrhizal walnut seedlings recorded higher mineral element concentrations, including P than non-mycorrhizal seedlings, regardless of soil water regimes (Behrooz et al., 2019). This shows the potential value of AMF for P acquisition by walnut trees. However, it is not clear whether and how the efficient AMF strain *D*. *spurca* affects P acquisition, leaf gas exchange, and sugar accumulation in walnut plants under soil P-deficient and P-sufficient conditions.

The purpose of this study was to analyze the effects of an efficient AMF strain (*D*. *spurca*) on growth and leaf P concentrations, gas exchange, chlorophyll component concentrations, sugar accumulation, and *PAP* and *PT* gene expressions in walnut seedlings grown in 1 and 100 μmol/L P levels.

## Materials and methods

### Plant culture

The seeds of walnut variety Liaohe No. 1 were provided by the Walnut Technology Extension Center of Baokang (Hubei, China). The seeds were surface disinfected with 75% ethanol for 8 min, washed with distilled water, soaked in distilled water for a week, and then sown in autoclaved sands for the germination in an incubator at 28°C/20°C (day and night temperature) and 80% relative humidity. The seedlings with four leaves were transplanted into plastic pots (2.4 L) pre-filled with hydrochloric acid-washing sand to reduce the interference of P in the substrate.

Based on the results of Huang et al. (2020), we selected the *D*. *spurca* strain as the fungal material because it showed relatively good effects on improving walnut growth. The *D*. *spurca* strain was isolated from the rhizosphere of tomato in Shouguang (Shandong, China). After the morphological identification, the strain of *D*. *spurca* was trapped by white clover under potted conditions. After approximately 11 weeks, the *D*. *spurca*-colonized roots and potted substrates were collected as the mycorrhizal fungal inoculums and stored at 4°C after natural air-drying. Before use, the spore density was 15 spores/g. The inoculation of *D*. *spurca* was carried out at the time of transplanting. A total of 120 g of mycorrhizal fungal inoculums was applied to the designed pot as the inoculation treatment. The equal amount of autoclaved mycorrhizal inoculums was applied to the uninoculated pot as the uninoculation treatment, followed by 2 mL of filtered (25 μm) solution with equal amount of mycorrhizal inoculums added to maintain the consistency of the microbiota except for the target strain.

Seven days after the inoculation, P treatments were applied. P concentrations in the potted substrate were achieved by controlling the KH_2_PO_4_ level in Hoagland nutrient solutions (pH 7.0), where 1 μmol/L and 100 μmol/L KH_2_PO_4_ was defined as the low P and moderate P level (Li et al., 2010). To reduce the difference in K levels of nutrient solutions among treatments, additional KNO_3_ was added to the P-deficient treatment to ensure the consistent K level. The nutrient solution was used at an intensity of 150 mL per pot at the three-day intervals.

All treated seedlings were placed in a greenhouse from May 4, 2020 to August 4, 2020, where environmental conditions were described in detail by Zou et al. (2021)

### Experimental design

This experiment consisted of two factors: one was inoculation with (+A) and without (-A) *D*. *spurca* and the other was P treatments with 1 μmol/L (P_1_) and 100 μmol/L (P_100_) P. Each treatment was replicated eight times in 32 pots, with one walnut seedling planted in each pot in a randomized arrangement.

### Determinations of variables

After three months of treatments, the plants were harvested. Plant height, stem diameter, and total biomass were measured directly before harvesting. At the same time, a portable Li-6400 photosynthetic apparatus (Li-Cor Inc., Lincoln, USA) was used to determine leaf gas exchange starting at 9:00 am on a sunny day before harvest. The soil attached to the roots was gently shaken off for hyphal length analysis, based on the method outlined by Bethlenfalvay and Ames (1987). A portion of root segments were cut, and root mycorrhizal staining was performed using trypan blue method described by Phillips and Hayman (1970). After microscopic observation, the root mycorrhizal colonization rate (%) was estimated as the percentage of the length of root segments colonized by AMF to the total length of root segments examined.

Eight plants from each treatment were divided equally into two parts, one of which was immediately frozen in liquid nitrogen and then stored at -80 °C for analysis of gene expressions. The other part was killed at 105 °C for 3 min after chlorophyll determination, then dried at 75 °C to constant weight, ground to powder, and passed through a 2 mm sieve for P concentration determination. The ICP Spectrometer (IRIS Advantage, Thermo, Waltham, USA) was used to analyze leaf P concentration. The concentration of glucose, fructose and sucrose in leaves was determined according to the colorimetric method described in detail by Wu et al. (2015b). The concentration of chlorophyll components was extracted with 80% acetone and determined using the method described by He et al. (2022).

The sequences of *PAP* genes (*PAP10* and *PAP12*) and *PT* genes (*PT3;1* and *PT3;3*) in Arabidopsis were obtained from the NCBI database (http://www.ncbi.nlm.nih.gov) and then compared with genome-wide of walnut (http://aegilops.wheat.ucdavis.edu/Walnut/data.php). The primer sequences (**Supplementary Table S1**) of *JrPAP10*, *JrPAP12*, *JrPT3;1*, and *JrPT3*;3 genes were designed using Primer5 premier 5.0 software and synthesized by Shanghai Bioengineering Co., Ltd. (Shanghai, China). Total RNA of leaf samples was extracted using an EASY spin Plus plant RNA kit (Aidlab). The reverse transcription of RNA was performed using the PrimeScript™ RT reagent kit with gDNA eraser kit (Takara). The 18S rRNA of walnut was used as the reference gene for qRT-PCR amplification. qRT-PCR was performed using the fluorescent dye method (2×AceQ^®^ qPCR SYBR^®^ Green Master Mix) with three biological replicates per treatment. The 2^-ΔΔCt^ method (Livak and Schmittgen, 2001) was used to calculate the relative gene expression, in which the relative expression was normalized by the treatment with non-inoculation of *D*. *spurca* at P_100_ levels.

### Statistical analysis

The data obtained from this experiment were presented using the means ± standard deviation. A two-factor (P treatments and AMF inoculations) analysis of variance was used for statistical analysis, with arcsine transformation preprocessed for percentages. Significant differences were compared at the 0.05 level using the Duncan’s new multiple range test. All statistical analyses were performed under SAS software.

## Results

### Changes in root AMF colonization and soil hyphal length

**Figure 1:**
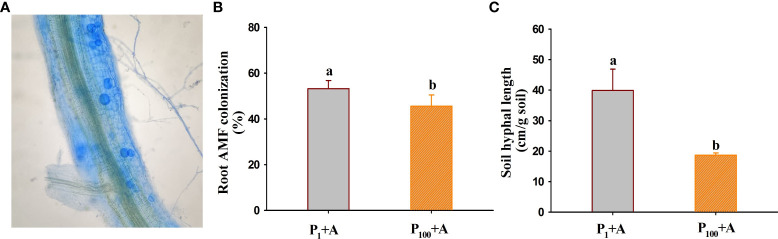
Root colonization **(A)** of *Diversispora spurca* and changes in root mycorrhizal colonization rate **(B)** and soil hyphal length **(C)** of walnut seedlings inoculated with *D*. *spurca* grown in 1 and 100 μmol/L phosphorus levels. Data (means ± SD, *n* = 4) followed by different letters above the bars indicate significant (*P* < 0.05) differences. P_1_+A, the walnut seedlings inoculated with *D*. *spurca* at 1 μmol/L phosphorus levels; P_1_-A, the walnut seedlings inoculated without *D*. *spurca* at 1 μmol/L phosphorus levels; P_100_+A, the walnut seedlings inoculated with *D*. *spurca* at 100 μmol/L phosphorus levels; P_100_-A, the walnut seedlings inoculated without *D*. *spurca* at 100 μmol/L phosphorus levels.

No mycorrhizal colonization was observed in roots of walnut seedlings inoculated without *D*. *spurca*, while mycorrhizal structures were visible in roots of walnut seedlings inoculated with *D*. *spurca* (**Figure 1A**), where the root mycorrhizal colonization rate ranged from 45.6% to 53.2% (**Figure 1B**) and soil hyphal length varied from 18.7 to 39.9 cm/g soil (**Figure 1C**), respectively. The P_1_ treatment significantly increased the root AMF colonization rate and soil hyphal length by 16.7% and 113.4%, respectively, compared with P_100_ treatment. P treatments and AMF inoculations significantly interacted with each other on soil hyphal length (**Table 1**).

**Table 1:** Significance in interaction between arbuscular mycorrhizal fungi (AMF) treatments and phosphorus (P) treatments.

	AMF treatments	P treatments	Interaction
Root mycorrhizal colonization rate	<0.0001	0.1234	0.1234
Soil hyphal length	<0.0001	0.0008	0.0008
Plant height	<0.0001	0.0016	0.1856
Stem diameter	0.0003	0.0004	0.8247
Total biomass	<0.0001	0.0005	0.1455
Chlorophyll *a*	0.1826	0.0015	0.6505
Chlorophyll *b*	0.1988	0.2294	0.2518
Carotenoid	0.1124	0.1108	0.9261
Total chlorophyll	0.1291	0.0038	0.4066
Leaf photosynthetic rate	0.0187	<0.0001	0.1631
Leaf transpiration rate	0.0162	0.0708	0.9161
Leaf stomatal conductance	0.0027	0.0341	0.9671
Leaf fructose	0.1184	0.8547	0.0036
Leaf glucose	0.3765	0.0185	0.0287
Leaf sucrose	0.5571	0.0009	0.1001
Leaf P	0.0029	0.0016	0.0540
Leaf *JrPT3;1*	<0.0001	<0.0001	<0.0001
Leaf *JrPT3;2*	0.0016	0.3693	0.0322
Leaf *JrPAP10*	<0.0001	<0.0001	<0.000
Leaf *JrPAP12*	<0.0001	<0.0001	0.0099

### Plant growth responses

**Figure 2:**
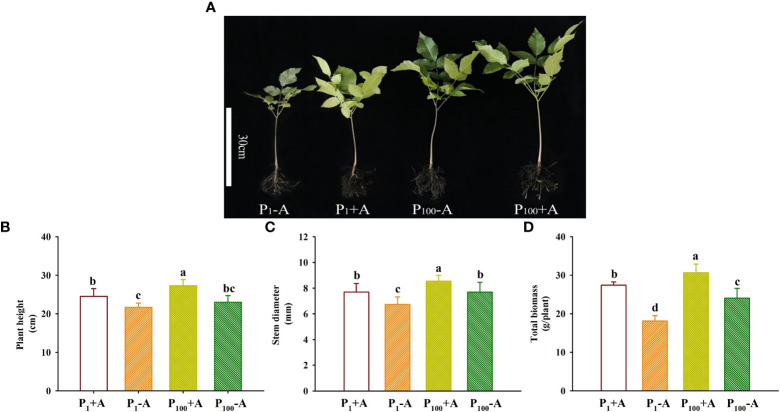
Changes in plant growth performance **(A)**, plant height **(B)**, stem diameter **(C)**, and total biomass production **(D)** of walnut seedlings inoculated with *Diversispora spurca* grown in 1 and 100 μmol/L phosphorus levels. Data (means ± SD, *n* = 4) followed by different letters above the bars indicate significant (*P* < 0.05) differences. The abbreviations were shown in **Figure 1**.

Walnut plants clearly responded to the P treatment as well as the *D*. *spurca* inoculation (**Figure 2A**). Both P treatments and AMF inoculations significantly altered plant growth variables, including plant height, stem diameter, and total biomass (**Figures 2B–D**). The P_100_ treatment significantly increased plant height, stem diameter, and total biomass by 6.0%, 14.1% and 32.9% in uninoculated plants and by 11.4%, 11.0% and 11.7% in inoculated plants, respectively, compared with the P_1_ treatment. On the other hand, inoculation with *D*. *spurca* also significantly increased plant height, stem diameter, and total biomass by 12.9%, 14.2%, and 51.5% under P_1_ conditions and by 18.7%, 11.2%, and 27.3% under P_100_ conditions, respectively, compared with the uninoculation control. There was not any significant interaction between AMF inoculation and P treatment on these growth variables (**Table 1**).

### Responses of leaf chlorophyll component concentrations

**Figure 3:**
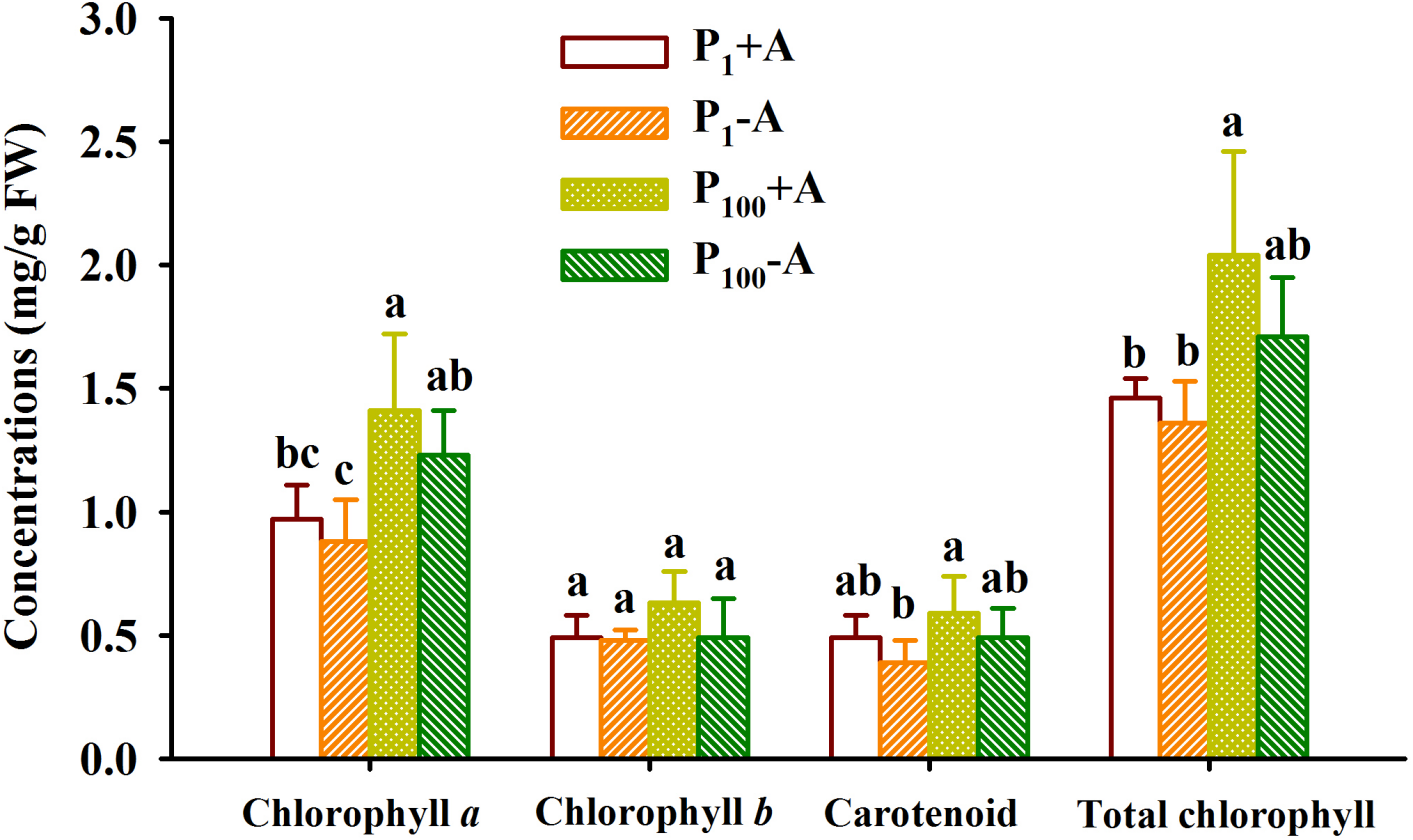
Changes in leaf chlorophyll component concentrations of walnut seedlings inoculated with *Diversispora spurca* grown in 1 and 100 μmol/L phosphorus levels. Data (means ± SD, *n* = 4) followed by different letters above the bars indicate significant (*P* < 0.05) differences. The abbreviations were shown in **Figure 1**.

P_100_ treatment significantly promoted concentrations of chlorophyll *a* in leaves of uninoculated plants by 39.8% as well as concentrations of chlorophyll *a* and total chlorophyll in leaves of inoculated plants by 47.4% and 39.7%, respectively, compared to P_1_ treatment (**Figure 3**). On the other hand, although walnut seedlings inoculated with *D*. *spurca* maintained relatively higher concentrations of chlorophyll *a*, chlorophyll *b*, carotenoid, and total chlorophyll, the difference was not significant, independent of substrate P levels. No significant interaction appeared on leaf chlorophyll component concentrations (**Table 1**).

### Responses of leaf gas exchange

**Figure 4:**
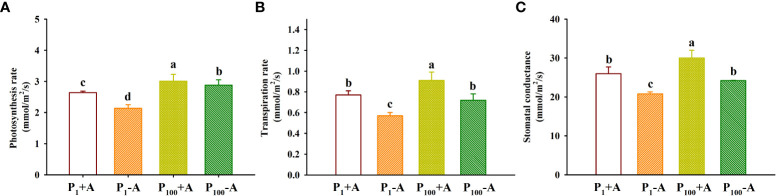
Changes in leaf photosynthesis rate **(A)**, transpiration rate **(B)**, and stomatal conductance **(C)** of walnut seedlings inoculated with *Diversispora spurca* grown in 1 and 100 μmol/L phosphorus levels. Data (means ± SD, *n* = 4) followed by different letters above the bars indicate significant (*P* < 0.05) differences. The abbreviations were shown in **Figure 1**.

The application of P_100_ significantly increased leaf photosynthetic rate, transpiration rate and stomatal conductance by 14.0%, 18.2%, and 15.4% in inoculated plants and 34.6%, 26.3%, and 16.3% in uninoculated plants, respectively, compared with P_1_ conditions (**Figures 4A–C**). Similarly, *D*. *spurca*-inoculated plants showed significantly higher leaf photosynthetic rate, transpiration rate, and stomatal conductance, with increases of 23.4%, 35.1%, and 25.0% under P_1_ conditions, and 4.5%, 26.4%, and 24.0% under P_100_ conditions, respectively. No significant interaction appeared on leaf gas exchange variables (**Table 1**).

### Responses of leaf sugars

**Figure 5:**
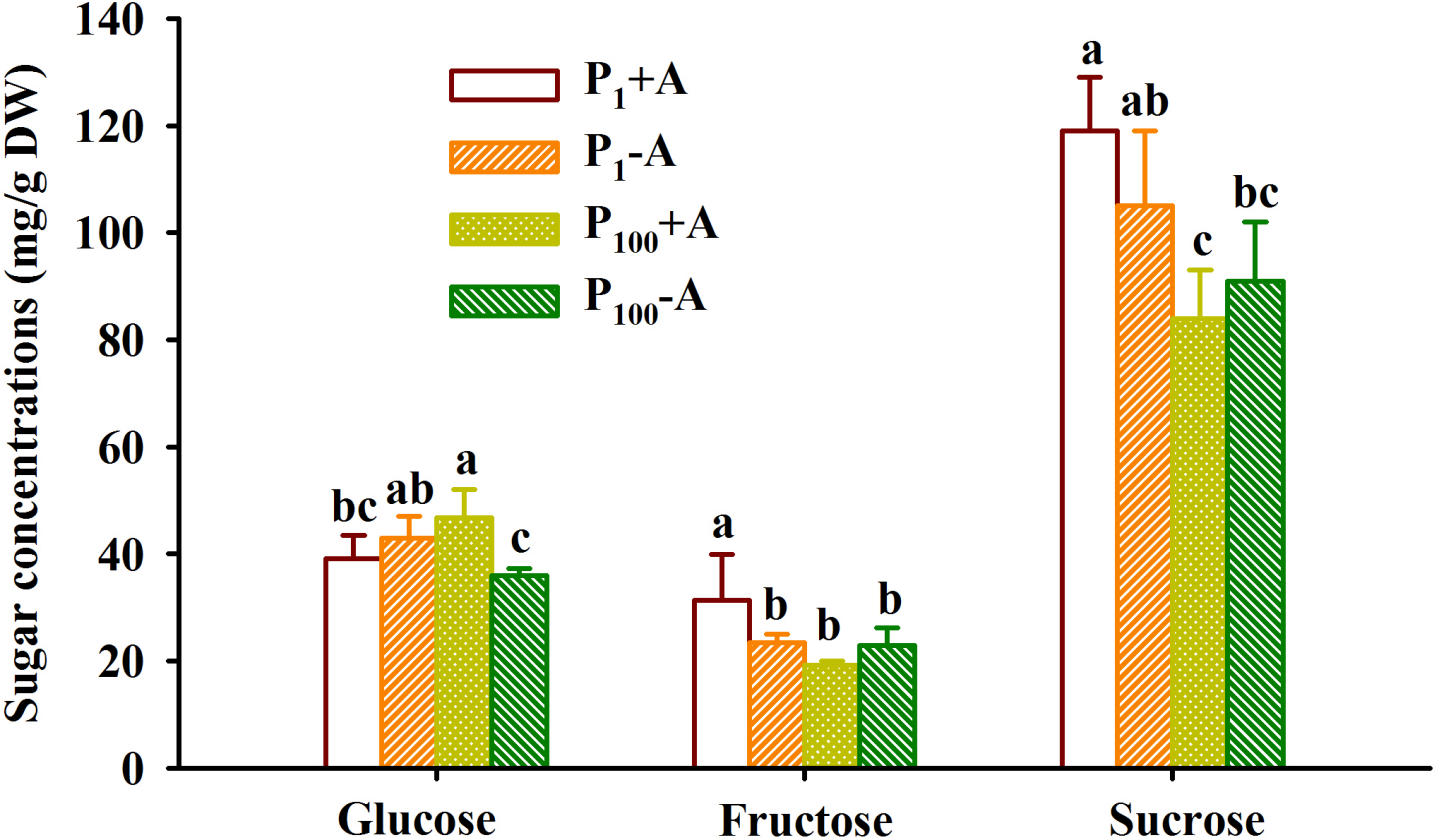
Changes in leaf fructose, glucose, and sucrose concentrations of walnut seedlings inoculated with *Diversispora spurca* grown in 1 and 100 μmol/L phosphorus levels. Data (means ± SD, *n* = 4) followed by different letters above the bars indicate significant (*P* < 0.05) differences. The abbreviations were shown in **Figure 1**.

P_100_ treatment dramatically reduced leaf glucose concentration of uninoculated plants, while distinctly increased leaf glucose concentration of inoculated plants by 19.7%, along with the reduction of leaf fructose and sucrose by 38.5% and 29.4%, respectively, compared with P_1_ treatment (**Figure 5**). On the other hand, mycorrhizal plants recorded 33.6% significantly higher leaf fructose concentration under P_1_ conditions and 30.0% significantly higher leaf glucose concentration under P_100_ conditions, respectively. In addition, AMF inoculation and P treatment significantly interacted to affect leaf fructose and glucose concentrations (**Table 1**).

### Responses of leaf P concentration

**Figure 6:**
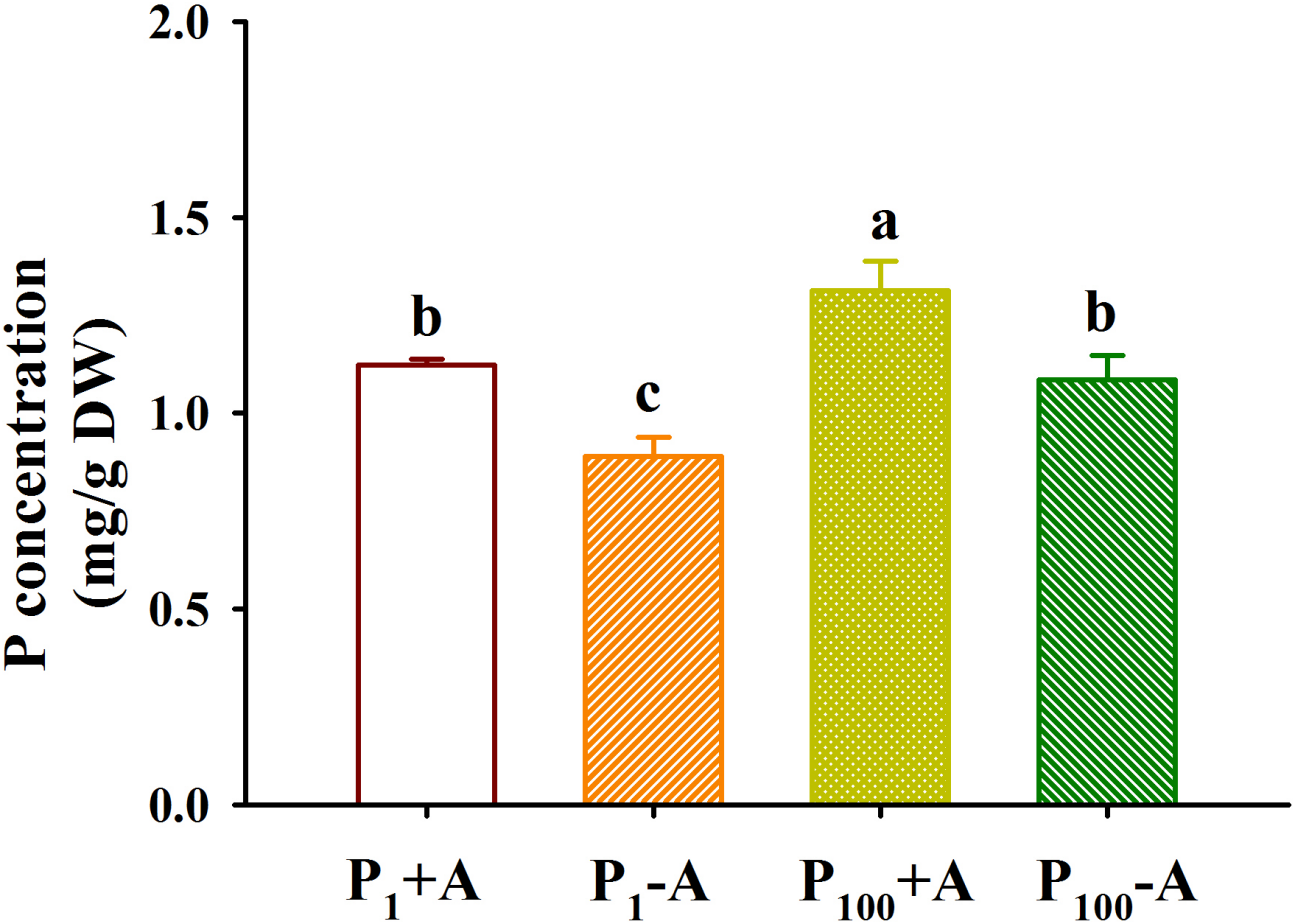
Changes in leaf phosphorus (P) concentrations of walnut seedlings inoculated with *Diversispora spurca* grown in 1 and 100 μmol/L P levels. Data (means ± SD, *n* = 4) followed by different letters above the bars indicate significant (*P* < 0.05) differences. The abbreviations were shown in **Figure 1**.

P_100_ treatment dramatically increased leaf P concentration by 22.1% in uninoculated plants and 17.1% in inoculated plants, respectively, compared with P_1_ treatment (**Figure 6**). In addition, *D*. *spurca* inoculation significantly increased leaf P concentration by 26.2% and 21.0% under P_1_ and P_100_ conditions, respectively. No significant interaction was found on leaf P concentration (**Table 1**)

### Responses of leaf PTs and PAPs expressions

**Figure 7:**
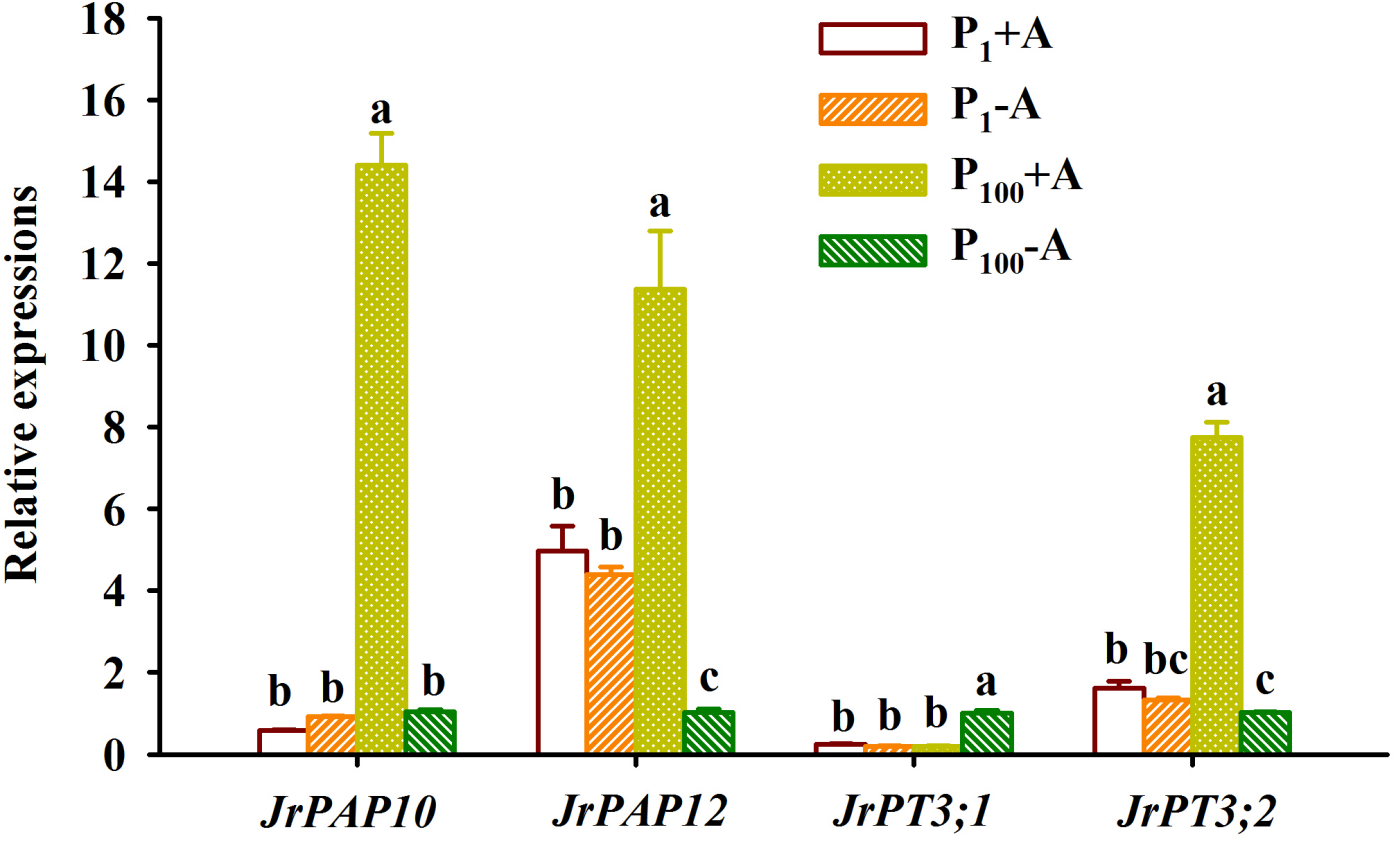
Changes in expressions of *purple acid phosphatase* (*PAP*) and *phosphate transporter* (*PT*) in leaves of walnut seedlings inoculated with *Diversispora spurca* grown in 1 and 100 μmol/L phosphorus levels. Data (means ± SD, *n* = 3) followed by different letters above the bars indicate significant (*P* < 0.05) differences. The abbreviations were shown in **Figure 1**.

P_100_ treatment dramatically up-regulated *JrPAP10*, *JrPAP12*, and *JrPT3;2* expressions in mycorrhizal plants by 23.48-, 1.29-, and 3.79-fold and *JrPT3;1* expression in non-mycorrhizal plants by 4.19-fold, along with a 0.77-fold down-regulated expression of *JrPAP12* in non-mycorrhizal plants, compared with P_1_ treatment (**Figure 7**). Under P_1_ conditions, *D*. *spurca* inoculation did not alter expressions of *JrPAP10*, *JrPAP12*, *JrPT3;1*, and *JrPT3;2*. However, under P_100_ conditions, the fungal inoculation up-regulated *JrPAP10*, *JrPAP12*, and *JrPT3;2* by 12.76-, 10.19-, and 6.59-fold, respectively, along with a 0.80-fold down-regulation of *JrPT3;1* expression, compared with non-inoculation control. AMF inoculations and P treatments significantly interacted with each other to affect leaf *JrPAP10*, *JrPAP12*, *JrPT3;1*, and *JrPT3;2* expression (**Table 1**).

## Discussion

The results of this study showed that *D*. *spurca* was able to establish mycorrhizal symbiosis with the roots of walnut seedlings. Low P (P_1_) treatment dramatically stimulated root AMF colonization rate and soil hyphal length, compared with moderate P (P_100_) treatment. This is consistent with the results of Wu et al. (2015b) inoculating *Funneliformis mosseae* on trifoliate orange at 0.5 and 50 μmol/L P levels, but contrary to the results of Shao et al. (2021) inoculating *Claroideoglomus etunicatum* on tea plants at 0.5 and 50 μmol/L P levels. Usually, AMF colonization of roots is negatively correlated with substrate P levels, and thus low P levels in substrates stimulate hyphal growth and mycorrhizal formation (Wu et al., 2015a). Moreover, in the condition of substrate P deficiency, plant growth is more dependent on the mycorrhizal pathway to obtain P (Smith and Smith, 2012). In the present study, low P treatment significantly reduced plant height, stem diameter, and biomass production of walnut seedlings, but inoculation with *D*. *spurca* alleviated the inhibitory effects of low P stress on plant growth to varying degrees, and the promoting effect of mycorrhizae on total biomass was higher under P_1_ conditions than under P_100_ conditions, indicating a more prominent role of mycorrhizae under low P conditions. In addition to the growth improvement of walnut seedlings by *D*. *spurca* under P stress, similar results occurred under soil drought conditions (Ma et al., 2022), showing the important role of the *D*. *spurca* strain on plant growth of walnut seedlings.

In this study, inoculation with *D*. *spurca* did not significantly alter concentrations of chlorophyll *a*, chlorophyll *b*, carotenoid, and total chlorophyll, independent of substrate P levels. However, in maize, inoculation with *Glomus mosseae* significantly increased chlorophyll levels at 0.05 and 1 mmol/L P levels (Feng et al., 2002), which was associated with the promotion of host Mg and Fe acquisition by AMF (Zhang et al., 2018). This showed that the improvement of chlorophyll component concentrations by AMF is variable and may be dependent on AMF, host plants, and environmental conditions. Mycorrhiza-triggered changes in chlorophyll compositions were not significant in walnut seedlings, which in turn caused leaf glucose, fructose, and sucrose contents to be barely affected at low and moderate P levels, although mycorrhizal walnut plants showed higher fructose concentrations under P_1_ conditions as well as glucose concentrations at P_100_. AMF-induced sugar changes of walnut seedlings were associated with mycorrhizal roots forming a strong carbon pool and greater mycorrhizal roots consumed a large amount of sugar (Wu et al., 2015b; He et al., 2022). More experiments are required to verify the reason. However, *D*. *spurca* inoculation significantly elevated leaf photosynthetic rate, stomatal conductance, and transpiration rate, although low P treatment inhibited leaf gas exchange. Thus, under low P stress, AMF may accelerate leaf gas exchange in host plants by reducing stomatal resistance as well as increasing transpiration fluxes (Zhu et al., 2011). Another explanation is that AMF alters the levels of endogenous hormones, especially abscisic acid, and thus regulates leaf gas exchange (He et al., 2019).

The present study showed that *D*. *spurca* inoculation significantly increased leaf P levels of walnut seedlings, and the increase was higher at P_1_ levels than at P_100_ levels, showing the prominent effect of *D*. *spurca* on host P uptake at low P levels. This is because plant roots at P_100_ levels can take up sufficient P and therefore have relatively low dependence on arbuscular mycorrhizae, whereas at P_1_ levels, plant roots do not take up sufficient P and need to take up additional P to meet plant P demand with the help of AMF through its extraradical hyphal extension to areas inaccessible to the root (Song et al., 2001).

PAP is the largest class of acid phosphatases, secreting acid phosphatase to the cell wall or to the root surface and environment, mainly involved in the hydrolysis of multiple Pi esters in the rhizosphere or extra-plasmic space, thus facilitating plant P acquisition (Schenk et al., 2013; Wang et al., 2014). The present study showed that in *D*. *spurca*-inoculated walnut seedlings, P_1_ treatment significantly suppressed the expression of both *JrPAP10* and *JrPAP12*, compared with P_100_ treatment, whereas in uninoculated plants, *JrPAP12* expression was up-regulated. This suggested that mycorrhizal plants are distinct from non-mycorrhizal plants in terms of *PAPs* expression response in the face of P stress. This is due to the fact that mycorrhizal plants exhibited higher mycorrhizal colonization rate of roots and soil hyphal length under low P versus moderate P conditions, and they can better help the host to obtain P through reducing arbuscule degradation (Li et al., 2019), resulting in a reduced dependence on *PAPs*. *D*. *spurca* did not alter *JrPAP10* and *JrPAP12* expression under low P conditions. However, at P_100_ levels, *D*. *spurca* significantly up-regulated *JrPAP10* and *JrPAP12* expression. Combined with no significant changes in *JrPT* expressions by *D*. *spurca* at low P levels, this implies that at low P levels, mycorrhizae promote plant P acquisition mainly by increasing root mycorrhizal colonization rate and soil hyphal length, which in turn directly exploits the mycorrhizal pathway to promote host P acquisition (Smith and Smith, 2011; Smith et al., 2015). In Arabidopsis, it has been demonstrated that *AtPAP10* is a secreted acid phosphatase and is an essential component of the adaptive response of plants to Pi limitation, but *AtPAP12* is an intracellular acid phosphatase (Wang et al., 2014). Therefore, at P_100_ levels, *D*. *spurca* might promote the expression of intracellular acid phosphatase (*JrPAP12*) and secret acid phosphatase genes (*JrPAP10*). In addition, *AtPAP10* on the root surface is controlled by ethylene only (Zhang et al., 2014), so whether mycorrhizal up-regulation of *JrPAP10* is related to mycorrhizal triggering of ethylene synthesis remains to be further investigated.

PTs are an important membrane protein found in the mitochondrial, plasma and plastid membranes of plants, controlling the uptake and translocation of Pi in plants (Nakamori et al., 2002). AMF usually induces the expression of host high-affinity *PT* genes to enhance plant uptake of P (Luo et al., 2019). The results of this study showed that low P treatment inhibited the expression of *JrPT3;1* in uninoculated plants as well as the expression of *JrPT3;2* in inoculated plants, suggesting that *JrPT3;1* and *JrPT3*;2 are not genes up-regulated for expression at low P levels. Huang et al. (2022) also found that low P repressed the expression of *PT1;1* in leaves of *Camellia oleifera* seedlings. However, walnut plants colonized by *D*. *spurca* did not show significant changes in *JrPT3;1* and *JrPT3;2* in leaves at low P levels, while at moderate P levels, *JrPT3;1* was repressed and *JrPT3;2* was up-regulated. This suggests that AMF only up-regulated the expression of *JrPT3;2* at appropriate P levels. In addition, mycorrhizal regulation of host *PTs* expression may be closely related to host root-hair status, where substrate low P levels increase root-hair density (Wang et al., 2006; Zhang et al., 2019; Shao et al., 2021), thereby promoting host P acquisition by both root-hairs and mycorrhizal pathways. Shao et al. (2021) also found that *Claroideoglomus etunicatum* up-regulated the expression of *CsPT1* but suppressed the expression of *CsPT4* in leaves of tea plants under low and appropriate P conditions. Cao et al. (2022) also found that *PTs* expressions were dependent on the AMF species and plant tissues. It concludes that AMF-up-regulated *PT* expressions depend on AMF species, plant tissues, substrate P levels, and *PT* genes. On the other hand, at moderate P levels, mycorrhizal enhancement of plant P acquisition is related to the up-regulation of host *PAPs* and *PTs* expression. More work needs to be done around the expression of more *PTs* genes in leaves and roots under mycorrhization conditions. Mycorrhizae can specifically induce the expression of certain high and low affinity *PT* genes (Rausch et al., 2001). In the present study, *JrPT3;2* may be a candidate *PT* gene specifically induced by AMF at moderate P levels, but functions of *JrPT3;2* in arbuscule-containing cortical cells of roots remains to be investigated.

## Conclusion

This study showed that low P treatment significantly inhibited plant growth, leaf gas exchange, chlorophyll component concentrations, and P concentration of walnut plants, but *D*. *spurca* significantly promoted plant growth and leaf gas exchange as well as P acquisition at low P levels. Although AMF did not induce the expression of *JrPAP10*, *JrPAP12*, *JrPT3;1*, and *JrPT3;2* at low P levels, the promotion of P acquisition by AMF at low P levels may be related to the increase of root AMF colonization and soil hyphal length. However, mycorrhizal promotion of host P acquisition at moderate P levels was associated with AMF-increased expression of *JrPT3;2*, *JrPAP10*, and *JrPAP12*. This suggests that AMF has different strategies to promote host P acquisition at different P levels. More studies need to be carried out on expressions of whole family members of *PTs* and *PAPs* in leaves and roots of walnut seedlings under mycorrhization conditions.

## Data availability statement

The original contributions presented in the study are included in the article/**Supplementary Material**. Further inquiries can be directed to the corresponding author.

## Author contributions

Y-NZ, Y-JX, and Q-SW designed the experiment. G-MH and R-CL prepared the materials for the experiment. G-MH and R-CL analyzed the data. Y-NZ and R-CL wrote the manuscript. AS, KK, AH, EFA, and Q-SW revised the manuscript. All authors contributed to the article and approved the submitted version.

## References

[B1] Adeyemi N. O. Atayese M. O. Sakariyawo O. S. Azeez J. O. Olubode A. A. Ridwan M.. (2021). Influence of different arbuscular mycorrhizal fungi isolates in enhancing growth, phosphorus uptake and grain yield of soybean in a phosphorus deficient soil under field conditions. Commun. Soil Sci. Plant Analy. 52, 1171–1183. doi: 10.1080/00103624.2021.1879117

[B2] Behrooz A. Vahdati K. Rejali F. Lotfi M. Sarikhani S. Leslie C. (2019). Arbuscular mycorrhiza and plant growth-promoting bacteria alleviate drought stress in walnut. HortScience 54, 1087–1092. doi: 10.21273/HORTSCI13961-19

[B3] Bethlenfalvay G. J. Ames R. N. (1987). Comparison of two methods for quantifying extraradical mycelium of vesicular-arbuscular mycorrhizal fungi. Soil Sci. Soc Am. J. 51, 834–837. doi: 10.2136/sssaj1987.03615995005100030049x

[B4] Cao M. A. Liu R. C. Xiao Z. Y. Hashem A. Abd Allah E. F. Alsayed M. F.. (2022). Symbiotic fungi alter the acquisition of phosphorus in *Camellia oleifera* through regulating root architecture, plant phosphate transporter gene expressions and soil phosphatase activities. J. Fungi 8, 800. doi: 10.3390/jof8080800 PMC9409677 36012789

[B5] Etesami H. Jeong B. R. Glick B. R. (2021). Contribution of arbuscular mycorrhizal fungi, phosphate–solubilizing bacteria, and silicon to p uptake by plant. Front. Plant Sci. 12, 699618. doi: 10.3389/fpls.2021.699618 34276750 PMC8280758

[B6] Feng G. Zhang F. Li X. Tian C. Tang C. Rengel Z. (2002). Improved tolerance of maize plants to salt stress by arbuscular mycorrhiza is related to higher accumulation of soluble sugars in roots. Mycorrhiza 12, 185–190. doi: 10.1007/s00572-002-0170-0 12189473

[B7] He J. D. Li J. L. Wu Q. S. (2019). Effects of *Rhizoglomus intraradices* on plant growth and root endogenous hormones of trifoliate orange under salt stress. J. Anim. Plant Sci. 29, 245–250.

[B8] He W. X. Wu Q. S. Hashem A. Abd Allah E. F. Muthuramalingam P. Al-Arjani A.-B. F.. (2022). Effects of symbiotic fungi on sugars and soil fertility and structure-mediated changes in plant growth of *Vicia villosa* . Agriculture 12, 1523. doi: 10.3390/agriculture12101523

[B10] Huang Y. X. Lin Y. L. Zhang L. P. Wu F. Yang Y. Tan M. X. (2022). Effects of AM fungi and inorganic phosphorus on phosphorus uptake and growth soil phosphorus fraction of *Camellia oleifera* seedlings. For. Res. 35, 33–41. doi: 10.13275/j.cnki.lykxyj.2022.005.004

[B9] Huang G. M. Zou Y. N. Wu Q. S. Xu Y. J. Kuča K. (2020). Mycorrhizal roles in plant growth, gas exchange, root morphology, and nutrient uptake of walnuts. Plant Soil Environ. 66, 295–302. doi: 10.17221/240/2020-PSE

[B13] Li Y. F. Jin S. H. Ye Z. Q. Huang J. Q. Jiang P. K. (2010). Root morphology and physiological characteristics in carya cathayensis seedlings with low phosphorus stress. J. Zhejiang A.&F. Univ, 27, 239–245.

[B12] Li X. R. Sun J. Albinsky D. Zarrabian D. Hull R. Lee T.. (2022). Nutrient regulation of lipochitooligosaccharide recognition in plants *via NSP1* and *NSP2* . Nat. Commun. 13, 6421. doi: 10.1038/s41467-022-33908-3 36307431 PMC9616857

[B11] Li C. C. Zhou J. Wang X. R. Liao H. (2019). A purple acid phosphatase, *GmPAP33*, participates in arbuscule degeneration during arbuscular mycorrhizal symbiosis in soybean. Plant Cell Environ. 42, 2015–2027. doi: 10.1111/pce.13530 30730567

[B14] Livak K. J. Schmittgen T. D. (2001). Analysis of relative gene expression data using real-time quantitative PCR and the 2^-ΔΔCt^ . Methods 25, 402–408. doi: 10.1006/meth.2001.1262 11846609

[B15] Luo Y. Y. Hao X. J. Zhang K. Y. (2019). Effect of inoculation of AM fungi on different phosphorus morphology of discarded soil in coal mining area. Southwest China J. Agric. Sci. 32, 381–388. doi: 10.16213/j.cnki.scjas.2019.2.026

[B16] Ma T. Ning D. L. (2021). Analysis of international competitiveness of China walnut industry. For. Sci. Technol. 64 (1), 3–7. doi: 10.13456/j.cnki.lykt.2019.12.26.0002

[B17] Ma W. Y. Qin Q. Y. Zou Y. N. Kuča K. Giri B. Wu Q. S.. (2022). Arbuscular mycorrhiza induces low oxidative burst in drought-stressed walnut through activating antioxidant defense systems and heat shock transcription factor expression. Front. Plant Sci. 13, 1089420. doi: 10.3389/fpls.2022.1089420 36523633 PMC9745176

[B18] Ma W. Y. Wu Q. S. Xu Y. J. Kuča K. (2021). Exploring *mycorrhizal fungi* in walnut with a focus on physiological roles. Not. Bot. Horti Agrobo. 49, 12363. doi: 10.15835/nbha49212363

[B19] Malhotra H. Vandana, Sharma S. Pandey R. (2018). “Phosphorus nutrition: Plant growth in response to deficiency and excess,” in Plant nutrients and abiotic stress tolerance. Eds. Hasanuzzaman M. Fujita M. Oku H. Nahar K. Hawrylak-Nowak B. (Singapore: Springer), 171–190. doi: 10.1007/978-981-10-9044-8_7

[B20] Mao J. H. Li R. B. Jing Y. B. Ning D. L. Li Y. P. Chen H. Y. (2022). Arbuscular mycorrhizal fungi associated with walnut trees and their effect on seedling growth. J. For. Environ. 42, 71–80. doi: 10.13324/j.cnki.jfcf.2022.01.009

[B21] Nakamori K. Takabatake R. Umehara Y. Kouchi H. Izui K. Hata S. (2002). Cloning, functional expression, and mutational analysis of a cDNA for *Lotus japonicus* mitochondrial phosphate transporter. Plant Cell Physiol. 43, 1250–1253. doi: 10.1093/pcp/pcf141 12407206

[B22] Phillips J. M. Hayman D. S. (1970). Improved procedures for clearing roots and staining parasitic and vesicular-arbuscular mycorrhizal fungi for rapid assessment of infection. Trans. Br. Mycol. Soc 55, 158–161. doi: 10.1016/S0007-1536(70)80110-3

[B23] Rausch C. Daram P. Brunner S. Jansa J. Laloi M. Leggewie G.. (2001). A phosphate transporter expressed in arbuscule-containing cells in potato. Nature 414, 462–465. doi: 10.1038/35106601 11719809

[B24] Sandhu J. Rouached H. (2022). All roads lead to PHO1. Nat. Plants 8, 986–987. doi: 10.1038/s41477-022-01242-7 36050465

[B25] Schenk G. Mitić N. Hanson G. R. Comba P. (2013). Purple acid phosphatase: a journey into the function and mechanism of a colorful enzyme. Coordin. Chem. Rev. 257, 473–482. doi: 10.1016/j.ccr.2012.03.020

[B26] Serr E. F. (1960). Walnut orchards on volcanic soils deficient in phosphorus. Calif. Agric. 14, 6–7.

[B27] Shao Y. D. Hu X. C. Wu Q. S. Yang T. Y. Srivastava A. K. Zhang D. J.. (2021). Mycorrhizas promote p acquisition of tea plants through changes in root morphology and p transporter gene expression. South Afr. J. Bot. 137, 455–462. doi: 10.1016/j.sajb.2020.11.028

[B28] Smith S. E. Anderson I. C. Smith F. A. (2015). Mycorrhizal associations and phosphorus acquisition: from cells to ecosystems. Annu. Plant Rev. 48, 409–440. doi: 10.1002/9781118958841.ch14

[B29] Smith S. E. Smith F. A. (2011). Roles of arbuscular mycorrhizas in plant nutrition and growth: New paradigms from cellular to ecosystem scales. Annu. Rev. Plant Biol. 62, 227–250. doi: 10.1146/annurev-arplant-042110-103846 21391813

[B30] Smith S. E. Smith F. A. (2012). Fresh perspectives on the roles of arbuscular mycorrhizal fungi in plant nutrition and growth. Mycologia 104, 1–13. doi: 10.3852/11-229 21933929

[B31] Song Y. C. Li X. L. Feng G. (2001). Effect of VAM fungi on phosphatase activity in maize rhizosphere. Chin. J. Appl. Ecol. 12, 593–596. 11758391

[B34] Wang Y. Chen Y. F. Wu W. H. (2021). Potassium and phosphorus transport and signaling in plants. J. Integr. Plant Biol. 63, 34–52. doi: 10.1111/jipb.13053 33325114

[B32] Wang L. Lu S. Zhang Y. Li Z. Du X. Liu D. (2014). Comparative genetic analysis of arabidopsis purple acid phosphatases AtPAP10, AtPAP12, and AtPAP26 provides new insights into their roles in plant adaptation to phosphate deprivation. J. Integr. Plant Biol. 56, 299–314. doi: 10.1111/jipb.12184 24528675

[B33] Wang Q. M. Peng W. X. Lu B. J. Pei D. (2006). Histological study of *in vitro* adventitious roots of *Juglans regia* . Acta Bot. Boreal.-Occident. Sin. 26, 719–724.

[B35] Wu Q. S. Li Y. Zou Y. N. He X. H. (2015a). Arbuscular mycorrhiza mediates glomalin-related soil protein production and soil enzyme activities in the rhizosphere of trifoliate orange grown under different p levels. Mycorrhiza 25, 121–130. doi: 10.1007/s00572-014-0594-3 25033923

[B36] Wu Q. S. Srivastava A. K. Li Y. (2015b). Effect of mycorrhizal symbiosis on growth behavior and carbohdyrate metabolism of trifoliate orange under different substrate p levels. J. Plant Growth Regul. 34, 495–508. doi: 10.1007/s00344-015-9485-x

[B37] Zhang C. P. (2014). “Influence of nitrogen or phosphorous on water metabolism of *juglans regia* seedlings,” in Dissertation for the doctoral degree (Chinese Academy of Forestry, Beijing, China).

[B39] Zhang T. Hu Y. Zhang K. Tian C. Guo J. (2018). Arbuscular mycorrhizal fungi improve plant growth of ricinus communis by altering photosynthetic properties and increasing pigments under drought and salt stress. Indust. Crops Prod. 117, 13–19. doi: 10.1016/j.indcrop.2018.02.087

[B40] Zhang Y. J. Sun F. Fettke J. Schottler M. A. Ramsden L. Fernie A. R.. (2014). Heterologous expression of *AtPAP2* in transgenic potato influences carbon metabolism and tuber development. FEBS Lett. 588, 3726–3731. doi: 10.1016/j.febslet.2014.08.019 25173632

[B38] Zhang F. Wang P. Zou Y. N. Wu Q. S. Kuča K. (2019). Effects of mycorrhizal fungi on root-hair growth and hormone levels of taproot and lateral roots in trifoliate orange under drought stress. Arch. Agron. Soil Sci. 65, 1316–1330. doi: 10.1080/03650340.2018.1563780

[B41] Zhu X. C. Song F. B. Liu S. Q. Liu T. D. (2011). Effects of arbuscular mycorrhizal fungus on photosynthesis and water status of maize under high temperature stress. Plant Soil 346, 189–199. doi: 10.1007/s11104-011-0809-8

[B42] Zou Y. N. Zhang F. Srivastava A. K. Wu Q. S. Kuča K. (2021). Arbuscular mycorrhizal fungi regulate polyamine homeostasis in roots of trifoliate orange for improved adaptation to soil moisture deficit stress. Front. Plant Sci. 11, 600792. doi: 10.3389/fpls.2020.600792 33510746 PMC7835339

