# Supplementary material for: Two different strategies of Diversispora spurca-inoculated walnut seedlings to improve leaf P acquisition at low and moderate P levels
Source: Front Plant Sci. 2023 Feb 23;14:1140467. doi: 10.3389/fpls.2023.1140467 (PMC9995707; doi:10.3389/fpls.2023.1140467)
Supplement: Supplementary file 1 [file DataSheet_1.pdf]

Supplementary Table S1 The primer sequence of selective genes in qRT-PCR

Genes	Accessions	Primer sequence (5’→3’)
JrPAP10	Jm3DG00060700	F: ACACCCCTCAATACCAATGGC
		R: ACTCTCATGGTTTCCCCCTCC
JrPAP12	Jm4DG00150700	F: ACACTCCACAATACAAATGGC
		R: TGGGCAATGCATAAAAACGAT
JrPT3;1	Jm2DG00002500	F: ATCTCATCTGGGTTTGGAGTG
		R: CTGAGCACTGTAGCCAAGCAA
JrPT3;2	Jm6DG00001600	F: TGCCCACTTTTCTAGGTTACA
		R: AGGTTTTGTACTTGGTTGCAT
18S rRNA	LOC109010173	F: GGTCAATCTTCTCGTTCCCTT
		R: TCGCATTTCGCTACGTTCTT
